# On the Use of Molecular Weight Cutoff Cassettes to Measure Dynamic Relaxivity of Novel Gadolinium Contrast Agents: Example Using Hyaluronic Acid Polymer Complexes in Phosphate-Buffered Saline

**DOI:** 10.1155/2011/808795

**Published:** 2011-11-16

**Authors:** Nima Kasraie, Henry Wayne Oviatt, Geoffrey David Clarke

**Affiliations:** ^1^Radiological Sciences, University of Texas Health Science Center at San Antonio, San Antonio, TX 78229, USA; ^2^Department of Chemistry, University of California-Irvine, Irvine, CA 92697-2025, USA

## Abstract

The aims of this study were to determine whether standard extracellular contrast agents of Gd(III) ions in combination with a polymeric entity susceptible to hydrolytic degradation over a finite period of time, such as Hyaluronic Acid (HA), have sufficient vascular residence time to obtain comparable vascular imaging to current conventional compounds and to obtain sufficient data to show proof of concept that HA with Gd-DTPA ligands could be useful as vascular imaging agents. We assessed the dynamic relaxivity of the HA bound DTPA compounds using a custom-made phantom, as well as relaxation rates at 10.72 MHz with concentrations ranging between 0.09 and 7.96 mM in phosphate-buffered saline. Linear dependences of static longitudinal relaxation rate (R1) on concentration were found for most measured samples, and the HA samples continued to produce high signal strength after 24 hours after injection into a dialysis cassette at 3T, showing superior dynamic relaxivity values compared to conventional contrast media such as Gd-DTPA-BMA.

## 1. Introduction

Gadolinium chelates of small molecular weight are extracellular space markers that improve tissue contrast by reducing the longitudinal relaxation time and enhancing the diagnostic efficacy of MRI for the detection and characterization of lesions and for the evaluation of perfusion and flow-related phenomena [1] and have been widely investigated structurally [2] and clinically [3] as MRI contrast agents. MRI blood-pool agents are often paramagnetic gadolinium chelates of high-molecular-weight polymers that are largely retained within the intravascular space during the timespan of an MRI scanning session [4]. 

It has been demonstrated that the relaxivity of a Gd(III) complex will increase upon slowing down its molecular tumbling rates [5] and synthesizing more rigid molecules [6], insofar as its water residence time is close to optimal [5]. Hyaluronic Acid (HA) is a natural polymer with a relatively stiff chain [7] and acid functional groups that can be utilized for coupling Gd contrast agents and has been investigated for use as a polymeric carrier for Gd complexes [8].

The final objective of this project was to develop a new class of contrast agents for imaging the blood pool based on HA-Gd complexes. The approach taken was to use standard extracellular contrast agents of Gd(III) ions in combination with a polymeric entity susceptible to hydrolytic degradation over a finite period of time, such as HA. The end goal of this study hence was to determine whether these MRI contrast agents have sufficient vascular residence time to obtain comparable vascular imaging to the currently approved compounds in use in Europe and the United States [9, 10] and to obtain sufficient data to show proof of concept that HA with Gd-DTPA ligands could be useful as vascular imaging agents.

In this exploratory study, the relaxivity of Gd-HA polymer complexes at 10.72 MHz was measured. We hypothesized that the bound Gd ion would be free to influence the R1 of extracellular water molecules, yet the molecular size of the Gd-polymer complex would have a molecular radius sufficiently large to prevent diffusion through the vascular wall. This would result in better vascular contrast at relatively low Gd concentrations over an extended time period. 

## 2. Methods 

### 2.1. Preparation of Samples

**Figure 1:**
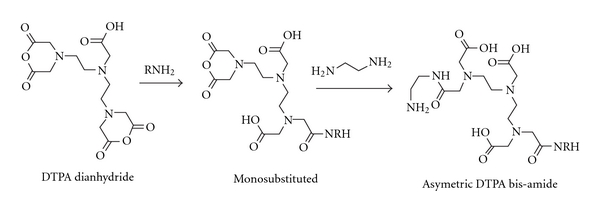
Scheme for mixed DTPA bisamide with free amine for coupling.

**Figure 2:**
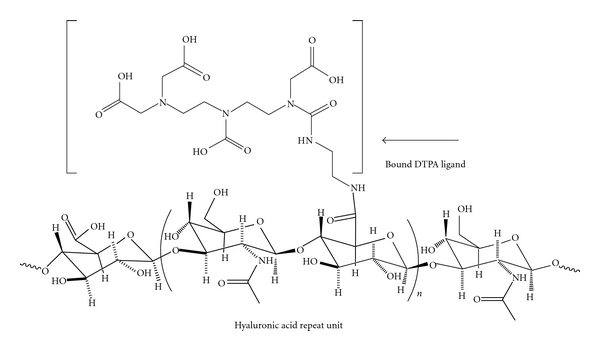
Hyaluronic acid repeat unit with amide bound diethylenediamine pentacetic acid. The Gd^3+^ ion is complexed with the bound DTPA ligand to yield the imaging agent, which is then coupled to HA via standard carbodiimide coupling methods.

We made DTPA dianhydride by the procedure of Geraldes et al. in pyridine and acetic anhydride at 65°C [11]. One can conceive of a number of approaches utilizing DTPA dianhydride with amines that result in bound DTPA ligands to an HA backbone. One approach is to couple ethylene diamine to the HA backbone by EDC methods, then to react the dianhydride with the amine functionalized HA. The second approach is first to react a monoamine with 1 : 1 stoichiometry with the dianhydride, then to open the second anhydride with the free amine of ethylenediamine bound to HA. A third approach is to vary the stoichiometry of the diamine and the dianhydride, essentially creating oligomeric DTPA ligands with free amine groups that can then be coupled via EDC methods to the HA backbone. These approaches using the dianhydride eliminate the deprotection steps of the Rappaport method [12], should reduce the amount of waste generated in the process, and more readily lend themselves to scale-up (i.e., it is a “greener” method). The samples used for imaging employed a 1 : 1 ratio of ethylene diamine to DTPA to make the DTPA amide from the DPTA dianhydride, which was then coupled to HA via the EDC/NHS method (Figures 1 and 2). Direct insertion probe Mass Spectrometry with negative ionization of the amine/DTPA oligomers was used to determine products in a crude form.

#### 2.1.1. Coupling of Gd-DTPA-Diamines to HA

Samples were prepared using diamine functionalized DTPA and coupled to HA solutions (0.5%) by way of standard EDC coupling methods in Hepes buffer to arrive at DTPA functionalized HA samples. Unhydrolyzed hyaluronic acid from Fluka with a high-molecular weight was used to determine the feasibility of the project concept. The retention of high-molecular weight HA derivatized with Gd complexes within a membrane material with a known low-molecular weight cutoff range was the expected result by virtue of the molecular dimensions' final contrast agent. For example, 10 mL of a 0.42% HA solution, 0.64 mls 0.1 M Hepes buffer at pH 7.0, 20 mg EDC, and 20 mg NHS were stirred for 30 min at room temperature, then 1.360 mL of the bisamine DTPA product (0.147 M in amine) previously complexed with GdCl_3_ in aqueous solution was added. 

#### 2.1.2. Determination of Complexed Gd Concentration and Relaxivities

**Figure 3:**
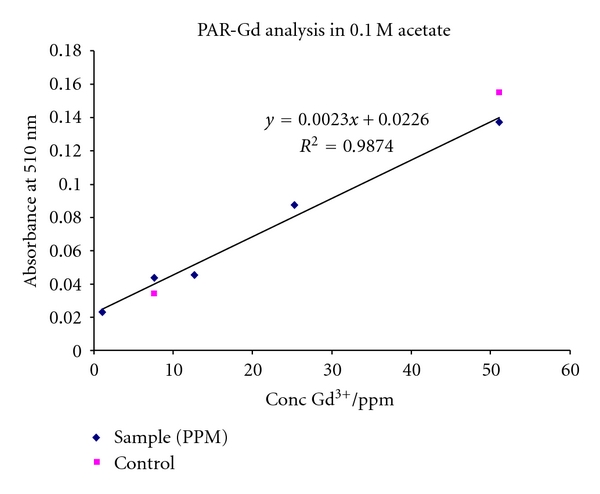
Pyridylazo resorcinol calibration curve used to determine Gd^3+^ concentrations in the samples tested for longitudinal relaxivity. The calibration curve was done using a solution of HA spiked with GdCl_3_ to simulate the desired matrix for analysis.

Concentration of Gd in the final HA complexes used for longitudinal static relaxivity measurements was determined photometrically by the method of Munshi and Dey by first ashing a sample in fuming nitric acid, followed by a second ashing in concentrated HCl to dryness, then redissolving the residue in distilled water volumetrically [13]. Aliquots of the final solution were used to determine the concentration of free Gd^3+^ with pyridylazo resorcinol (PAR) to determine the concentration of HA-DTPA-Gd complexes from a calibration curve (Figure 3). 

The concentration information was then used to dilute samples to measure the longitudinal relaxation rates of the complexes as a function of concentration. To perform these measurements, dried quantities of the HA-Gd-DTPA end samples were mixed into Dulbecco Phosphate Buffered Saline in small tubes (2.1 × 8.0 cm) to obtain final concentrations of 0.09, 0.12, 0.14, 0.17, 0.27, 0.50, 0.51, 0.62, 0.73, 0.95, 1.00, 1.26, 1.37, 1.54, 1.59, 1.70, 1.78, 2.21, 2.50, 2.53, 5.00, 6.32, and 7.96 mM. Although limited in choice, these concentrations were chosen so as to be in proximity of the range for clinical relevance. 

 The final prepared sample, called HWO-147, was an unhydrolysed hyaluronic acid DTPA complex whose concentration of Gd-DTPA was about 10% by weight. To prepare the samples for relaxation measurements, the desired amount of sample was immersed in buffered saline after cutting the freeze-dried samples with a razor blade on a clean surface. They were then kept at 3°C overnight to hydrate and stirred the following morning to obtain a homogenous state. The samples were kept at that temperature to minimize hydrolysis, until immediately before the measurements the following day, at which time, the samples were heated to 39°C, the temperature inside the coil, using a water bath.

The instrument used to measure the T1 relaxation time of all samples was a tabletop 10.72 MHz NMR system (PR-103 NMR Analyzer, PRAXIS, San Antonio, Tex, USA) designed specifically for performing relaxometry measurements on small samples. 

The R1 relaxation rates were measured in volumes of 7 mL (to receive the coil's maximum signal), and static r1 relaxivities were determined by estimating the slope of the relaxation-concentration fitted curve for each type of solution. 

### 2.2. Gd-HA Vascular Diffusion Imaging

In order to show that the agent has a sufficiently high blood-pool residence time, a selected sample from the measured solutions was examined to see if the hydrodynamic size of the Gd-complex polymer conjugate would be large enough to prevent immediate diffusion through vascular wall-like conditions. Three mL of HWO1-147 at a concentration of 2.5 mM in 10 mL of PBS was injected into a 10,000 Dalton molecular weight cutoff (MWCO) cassette (Slide-A-Lyzer, Thermo Fisher Scientific, Waltham, Mass, USA). A second cassette was filled with an extracellular MR contrast medium; Gd-DTPA-BMA (Omniscan, GE Healthcare, Chalfont St. Giles, UK) for comparison. A phantom was then constructed by simultaneously attaching two separate containers positioned horizontally to the transmit/receive 3T head coil in the MRI system. The two cassettes were each affixed to the containers separately while being immersed inside a 500 mL beaker of Dulbecco Phosphate buffered saline; a volume that was large enough to avoid any potential saturation effects in the solution. 

The phantoms were imaged simultaneously at 8 intervals over a period of 24 hours at room temperature. The MR image was acquired using a 3 Tesla MRI system (Magnetom Trio, Siemens, Malvern, Pa, USA). A T1-weighted spin echo inversion recovery sequence was used in which TE = 17 ms, TR = 750 ms, NSA = 2, FOV = 200 × 120 mm, slices = 5, and the matrix size was 200 (FE) × 120 (PE). A TI of 300 ms was used to maximize the signal-to-noise ratio. In between MRI scans, fluid circulation was set up within each container using stir bars to ensure a homogenous distribution of contrast in the containers.

### 2.3. Image Analysis

The obtained images in the phantom experiment were intended to be analyzed visually, as a qualitative measure. However in order to obtain some degree of quantifiable information on the signals, the *characteristic time* of the signal drop for each type of contrast agent was also estimated, that is, the time it takes for a given quantity to drop to 1/e (37%) of its initial value. To estimate this quantity, the center slices of each scan (8 in total) were selected, the signal from an ROI placed at the center of each cassette was consistently measured, and the background (in the container) with the same area was then similarly recorded for all 8 points. We then plotted the signal intensity for a fixed ROI against time (with *t* = 0 being the injection point) on semilog graph, and calculated the magnitude of the linearly fitted slope. 

## 3. Results

**Figure 4:**
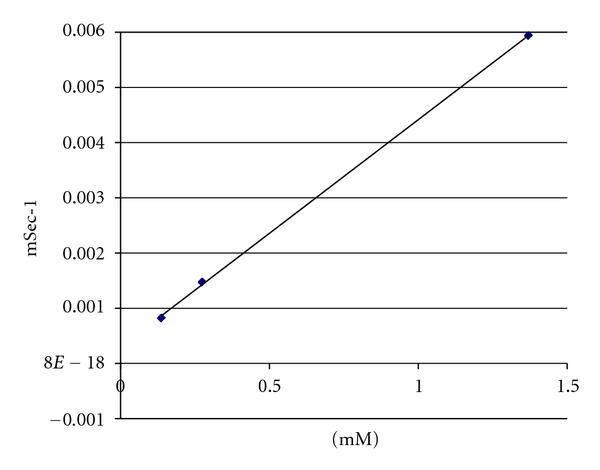
One of several experimental trials for determining the relaxometry properties of HA-DTPA-Gd complexes. The Relaxivity was calculated by estimating the slope of the relaxation rates (R) as a function of concentration (mM).

The relaxivity value of the measured HA conjugates at concentrations ranging from 0.09 mM to 7.96 mM and the corresponding standard error estimate using a commercial graphing program (SigmaPlot v7.0, Systat Software Inc., Chicago, Ill, USA) was 4.12 ± 0.07 (mM·s)^−1^ for HWO-147. The uncertainty of the relaxivity was calculated as the mean of the absolute value of the difference between each data point and the curve fitted to the relaxation data (Figure 4). 

As a comparison, the corresponding measured relaxivity for Gd-DTPA-BMA was 4.90 ± 0.01 (mM·s)^−1^. The manufacturer (Nycomed Ireland Ltd, IDA Industrial Estate, Carrigtwohill, Cc. Cork Ireland) reports a value of 4.6 mM^−1 ^s^−1^ obtained at 10 MHz field strength at 37 degrees, and current commercially available contrast agents have relaxivities ranging from 4.5 s^−1^mM^−1^ at 10.64 MHz to 3.5 s^−1^mM^−1^ at 85.14 MHz [14]. 

**Figure 5:**
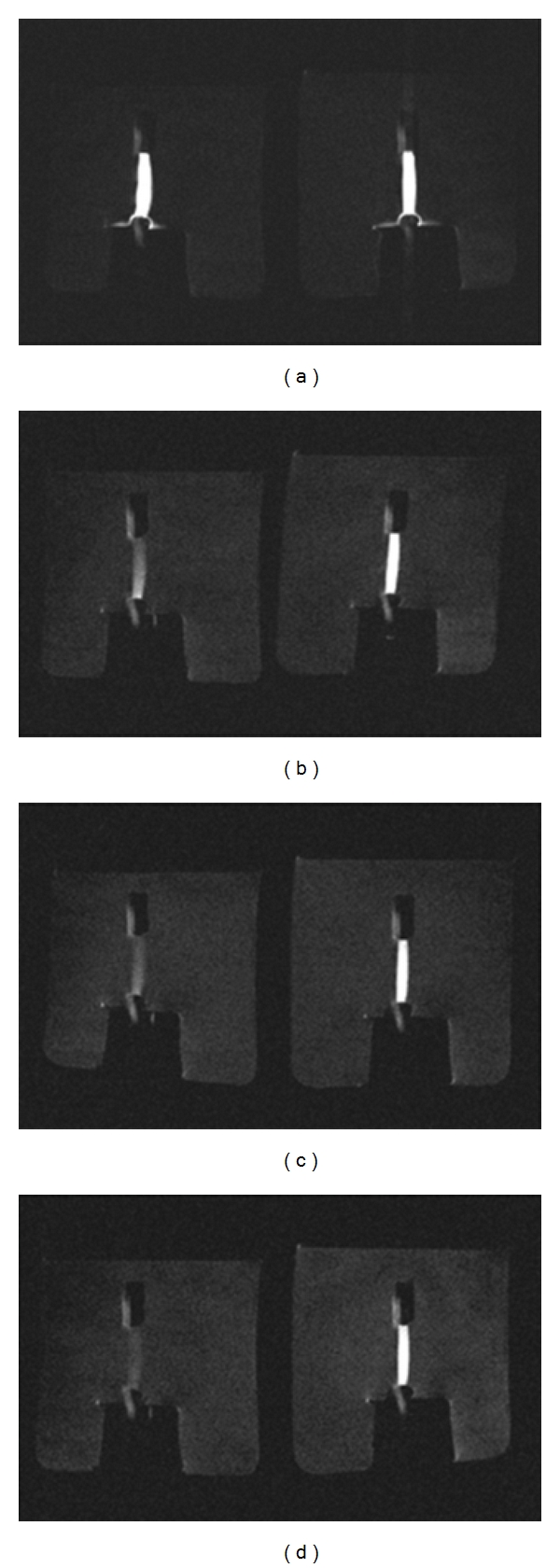
Magnetic resonance image montage of the progression of contrast media diffusion through a dialysis cassette at *t* = 0, 329, 407, and 1489 minutes after injection, starting from top-left row, ending at bottom right. At 24 hours, the Gd DTPA-BMA (left fixture) has leaked out, while the HWO1-147 (right fixture) is relatively well-retained inside its cassette.

Figure 5 shows the 24 hour progression of the phantom after injection, with Gd-DTPA-BMA having extensively leaked out of the cassette, while the HWO1-147 cassette has relatively retained its contents. 

**Figure 6:**
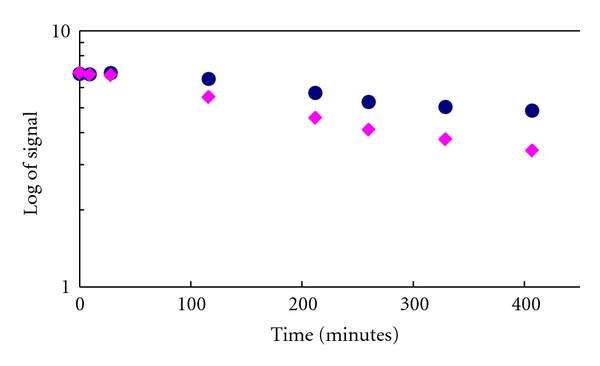
MRI intensity drop of Gd DTPA-BMA versus HWO1-147 plotted against time. The semi-log plot is used to estimate the characteristic time of the signal drop for Gd DTPA-BMA (pink diamonds) versus HWO1-147 (dark circles), by calculating the magnitude of the slope for each medium.

The semi-log slope to the fitted plots shown in Figure 6 was negative as expected, and in agreement with visual inspection of T1 images. The plots also indicate that at the time of injection (*t* = 0), both cassette signals are of almost equal strength, while at 400 minutes after injection, the retention of the HWO1-147 signal strength can be clearly deduced by the differing slopes of the two samples. Their estimated characteristic times were 706.93 min for HWO1-147 and 429.23 min for Gd-DTPA-BMA.

## 4. Discussion 

In this exploratory study, we hypothesized that the effective hydrodynamic volume of the contrast agent by linking a standard Gd-DTPA contrast agent to HA decreased the diffusion of the complex across a semipermeable dialysis membrane as a model of the vascular walls. This was shown by the ability of a dialysis membrane with a molecular weight cutoff of 10 kD to retain the coupled contrast agent for over twenty-four hours in an aqueous solution. Wen et al. have previously used 10 kD cassettes for the characterization and preliminary evaluation of Gd^3+^-chelated poly(L-glutamic acid) polymers for blood-pool imaging applications [15]. The MWCO must be high enough to allow passage of blood proteins and nutrients, but low enough to reject the blood-pool agent. Our results showed that the highest dynamic relaxivity was realized in the oligomeric complexes of Gd coupled to the HA backbone. Among these, sample HWO1-147 demonstrated the closest static relaxivity to Gd-DTPA-BMA, albeit being an unfractionated sample.

We used the procedure of Geraldes et al. to make DTPA dianhydride in pyridine and acetic anhydride at 65°C [11]. A significant advantage from a clinical point of view of an anhydride synthesis approach is that these ligands have only three free carboxylates (and a charge of 3−) at physiological pH. When complexed with Gd^3+^, they result in a neutral species.

Whereas an increase in the potential blood-pool residence time was observed in this study, it is anticipated that the development of improved MRI blood-pool contrast agents utilizing Hyaluronate polymers may lead to increased utility of MRI methods for the assessment of tissue blood perfusion, tissue blood volume, and quantitative microangiography. The primary anticipated application of these reagents would be that the attachment of semirigid macromolecules to the Gd complexes would improve both the residence time and signal enhancement in blood-pool MRI imaging. 

Various polymeric MRI contrast agents, including human serum albumin, polylysine, dendrimers, polyamide, and grafted copolymers, have been synthesized and evaluated as blood-pool imaging agents [15]. The clinical applications of many current macromolecular contrast agents remain limited due to toxicity considerations [15]. By utilizing DTPA coupling methods that would give a direct ester link of the DTPA ligand to HA, the hydrolysis product of this adduct would result in an HA molecule and free DTPA-Gd, which is already an FDA approved product. Therefore with this methodology we did not anticipate any significant toxicity from the DTPA-Gd complex over that of approved products. 

Furthermore, in order to fully investigate the efficacy of these contrast media, future studies should be conducted at multiple frequencies to obtain and examine their NMRD profiles, as they are a useful tool for studying the proton relaxation enhancement of MRI contrast agents [16].

## 5. Conclusion

In this study, we hypothesized that HA-Gd complexes have the potential for extended lifetime in the blood pool, which is potentially useful for magnetic resonance angiography applications over conventional Gd(III) chelates. We used static and dynamic relaxivity measurements to examine whether HA polymeric chelates would reside in the blood long enough to allow for thorough MRI examinations, using a semi-permeable dialysis membrane as a model of vascular walls. The synthesized HA compounds showed superior dynamic relaxivity values compared to conventional contrast media such as Gd-DTPA-BMA.

## Abbreviations

BMA: Bismethylamide
DTPA: Diethylene triamine pentaacetic acid
EDC: 1-Ethyl-3-(3-Dimethylaminopropyl)carbodiimide Hydrochloride
FE: Frequency Encoding
FOV: Field of View
Gd: Gadolinium
NHS: N-hydroxy succinimide
NMRD: Nuclear Magnetic Relaxation Dispersion
NSA: Number of Signal Averages
PE: Phase Encoding
R1: Longitudinal relaxation rate
TE: Time to Echo
TI: Inversion Time
TR: Repetition Time.
